# Contribution of Exogenous Proline to Abiotic Stresses Tolerance in Plants: A Review

**DOI:** 10.3390/ijms23095186

**Published:** 2022-05-06

**Authors:** Marjanossadat Hosseinifard, Szymon Stefaniak, Majid Ghorbani Javid, Elias Soltani, Łukasz Wojtyla, Małgorzata Garnczarska

**Affiliations:** 1 Department of Agronomy and Plant Breeding Sciences, College of Aburaihan, University of Tehran, Tehran P.O. Box 3391653755, Iran; marhos1@st.amu.edu.pl (M.H.); mjavid@ut.ac.ir (M.G.J.); elias.soltani@ut.ac.ir (E.S.); 2 Department of Plant Physiology, Institute of Experimental Biology, Faculty of Biology, Adam Mickiewicz University, ul. Uniwersytetu Poznańskiego 6, 61-614 Poznań, Poland; szymon.stefaniak@amu.edu.pl (S.S.); garnczar@amu.edu.pl (M.G.)

**Keywords:** drought stress, exogenous application, foliar spray, osmoprotectants, salinity stress, seed priming

## Abstract

Abiotic stresses are the major environmental factors that play a significant role in decreasing plant yield and production potential by influencing physiological, biochemical, and molecular processes. Abiotic stresses and global population growth have prompted scientists to use beneficial strategies to ensure food security. The use of organic compounds to improve tolerance to abiotic stresses has been considered for many years. For example, the application of potential external osmotic protective compounds such as proline is one of the approaches to counteract the adverse effects of abiotic stresses on plants. Proline level increases in plants in response to environmental stress. Proline accumulation is not just a signal of tension. Rather, according to research discussed in this article, this biomolecule improves plant resistance to abiotic stress by rising photosynthesis, enzymatic and non-enzymatic antioxidant activity, regulating osmolyte concentration, and sodium and potassium homeostasis. In this review, we discuss the biosynthesis, sensing, signaling, and transport of proline and its role in the development of various plant tissues, including seeds, floral components, and vegetative tissues. Further, the impacts of exogenous proline utilization under various non-living stresses such as drought, salinity, high and low temperatures, and heavy metals have been extensively studied. Numerous various studies have shown that exogenous proline can improve plant growth, yield, and stress tolerance under adverse environmental factors.

## 1. Introduction

Plants during their life cycles are exposed to various abiotic stresses such as salinity, drought, high/low temperatures, heavy metal toxicity, flooding, UV-B radiation and ozone which have adverse effects on growth, productivity and quality [1]. Abiotic stresses can reduce plant yield by 50 to 70% [2]. According to FAO 2021, study undergoing from 2008 to 2018 showed that drought stress alone lead to decrease in agriculture production over 34% what contributed to loss 37 billion USD and has been the most destructive abiotic stress on crop yields [3]. Therefore, limiting the decline of agricultural production under stress is a huge humanity challenge to ensure food security. Several different attempts have been developed to minimalize the negative effect of abiotic stresses on plant productivity like breeding programs to introduced new cultivars, genetic engineering, CRISPR/Cas9 technology, as well as plant stimulation by application exogenous molecules to plants [4]. Various strategies have been proposed to improve production in stressed plants. In recent years, the exogenous application of phytoprotectants has been found effective in mitigating the damages caused by stress in plants. Microbial origin phytoprotectants (arbuscular mycorrhiza and Rhizobacteria), nitrogen-containing phytoprotectants including osmoprotectants (amino acids, betaines and polyamines), melatonin, phytohormones (abscisic acid, salicylic acid, gibberellic acid, jasmonic acid and brassinosteroids), antioxidants (ascorbic acid, glutathione, tocopherol), redox signals (hydrogen peroxide, nitric oxide), micro nutrients (iron and zinc) and trace elements (selenium and silicon) are examples of phytoprotectants [5,6]. The application of osmoprotectants has attracted a lot of attention due to their high efficiency, ease of use, low cost, and no need for advanced equipment. Osmoprotectants or compatible solutes are small, highly soluble organic molecules at physiological pH with a neutral charge and low toxicity [7]. According to the DEOP database, 143 osmoprotectant compounds were identified [8]. Osmolytes are classified into three main groups including sugars and sugar alcohol (e.g., trehalose and inositol), ammonium compounds (e.g., glycine betaine and polyamines), and amino acids (e.g., proline and ectoine) [9]. Physiological, biochemical, and molecular studies in vivo and in vitro show that osmolites play important roles in the plant’s defense system against stress [10]. In the stress conditions, osmolytes accumulation in the plant cells uptakes water by osmotic adjustment and maintains cellular turgor [7].

Proline (Pro) is known as one of the most effective osmoprotectants and signaling molecules due to its cyclic structure and a secondary amino group (the α-amino group) distinct from the proteinogenic amino acids. Also, it has an essential role in primary metabolism in the forms of free amino acids and as part of proteins [10,11]. Much evidences suggest the positive correlation between the accumulation of proline and improving stress tolerance in the plants [12,13,14]. Proline accumulates in the cytosol without damaging cellular structures and it is a crucial part of the physiological adaptation in many plant species to stress [15,16]. Proline can improve protein stability and protects membranes integrity by binding to hydrogen bonds [7]. Also, proline may protect cells by increasing water uptake potential and facilitating activation of enzymes [11]. Despite acting as an osmolyte, proline is also considered as a potent antioxidative defense molecule, a metal chelator, a protein stabilizer, a ROS scavenger, and an inhibitor of programmed cell dying [16,17]. Abundant evidences suggest that the use of exogenous proline can increase the stress tolerance of plants [18,19,20]. Such application may trigger stress prevention mechanisms by contributing to stress tolerance mechanisms and an enhanced response to stress triggers during a subsequent stressful condition [21]. Genetic and biochemical modifications taking place in the plant after stress exposure and bringing up the reactions to the next stress are described as plant stress imprints [22] that could be induced by priming including plant treatment with molecules accumulated in response to stress. There are several methods for applying exogenous proline, the most important of them are foliar spraying and seed priming. The present review summarizes the current state of knowledge on the methods of exogenous proline application such as seed priming, foliar spraying, culture medium, seedling treatment, irrigation, etc. with a general note on biosynthesis, signaling and transportation, and the role of proline in abiotic stress. In this review, we also discuss the useful effects of exogenous proline application on plants exposed to different stress situations. To understand the diversity and commonality of responses, known and recently described examples in plant species were reviewed.

## 2. Proline Biosynthesis

Biosynthesis, catabolism, and transport between cells and different compartments of cells control intracellular proline levels in plants [23]. TSo far, pyrroline-5-carboxylate (P5C) is the only confirmed precursor for proline biosynthesis in plants. In prokaryotes, three enzymes are involved in proline biosynthesis, including ornithine cyclodeaminase (OCD), pyrroline-2-carboxylate reductase (P2CR) and delta-1-pyrroline-5-carboxylase reductase (P5CR) [7]. Multiple enzymes regulate the pathways involved in proline synthesis and degradation [10]. In plants under stress conditions, the accumulation of proline in addition to increased synthesis is due to the inactivation of degradation pathways [24]. The accumulation of proline in the cell is due to the activation of delta-1-pyrroline 5-carboxylase synthetase (P5CS) and P5CR, and also inhibition of proline oxidizing enzymes such as pyrroline dehydrogenase (Pro DH) and delta-1-pyrroline 5-carboxylase dehydrogenase (P5CDH).

**Figure 1:**
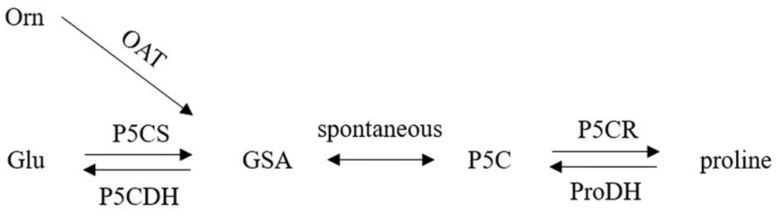
The metabolic pathway of proline synthesis in plants through glutamate and ornithine. Orn (represents ornithine), OAT (ornithine aminotransferase), Glu (glutamic acid), P5CS (delta-1-pyrroline-5-carboxylate synthase), P5CDH (delta-1-pyrroline-5-carboxylate dehydrogenase), GSA (glutamate-1-semialdehyde), P5C (pyrroline-5-carboxylate), P5CR (pyrroline-5-carboxylate reductase), ProDH (proline dehydrogenase).

Biosynthesis of proline in plants takes place in the chloroplast and cytoplasm, whereas proline catabolism occurs in mitochondria [25]. Proline synthesis has two pathways in plants and both glutamic acid (Glu) and ornithine (Orn) are precursors of proline biosynthesis [23] (Figure 1). Glu pathway is the main pathway to synthesize proline and Orn pathway is considered an alternative pathway based on radiotracer studies, co-expression analyses and analogy to mammals [26]. It has been reported that the ornithine pathway occurs during supra-optimal nitrogen conditions and seedling development. However, the Glu pathway becomes the main pathway to synthesize proline, under nitrogen-deficient and/or other stresses [24,27]. Proline biosynthesis from Glu occurs in the cytoplasm and/or chloroplast via two enzymatic steps. Glutamic acid (Glu) is reduced to glutamate-1-semialdehyde (GSA) by P5CS and then converted to P5C. Next, the second enzyme, P5CR can reduce P5C to proline. The electrons needed for this process are supplied by two NADPH and one ATP is consumed in proline biosynthesis [23,28].

In most plant species, the first step of proline biosynthesis is the rate-limiting step of proline. This is because several factors, including ABA, epigenetic modifications, and alternative splicing can regulate *P5CS* expression. Two isoforms of P5CS catalyse the first step of proline biosynthesis from glutamate *P5CS* [27]. While P5CR is encoded by only one gene in most plant species, in *Arabidopsis* two genes are involved in the formation of the P5CS enzyme. *P5CS1* is induced by stress known as the main contributor to stress-proline accumulation. While P5CS2 is considered to be important for embryonic development and growth [29]. Catabolism of proline back to glutamate also occurs in the mitochondria also via two enzymatic steps and both pro-biosynthesis and catabolism use the common intermediate P5C [16]. In proline catabolism, proline is converted to P5C by proline dehydrogenase or proline oxidase (PDH or POX) and P5C dehydrogenase (P5CDH) producing glutamate from P5C. While two genes encode PDH, a single *P5CDH* gene has been identified in *Arabidopsis* and tobacco [30,31]. Proline degradation is prevented during abiotic stress, as PDH transcription is activated by rehydration but suppressed by dehydration [15].

## 3. Proline Sensing, Signaling and Transportation

Proline has different roles in plants during growth and development and exposure to biotic and abiotic stresses. Thus, proline content and its transportation in plants are essential for crop production in higher quality and quantity. Under abiotic stresses, proline transport depends on environmental signals [28]. Proline metabolism in plants has been investigated for more than 40 years but little is known regarding the signaling pathways [15]. Duo to proline metabolism, Intracellular transport of proline takes place between cytosol, chloroplasts, and mitochondria. Proline transport and its amount in different tissues like xylem and phloem are influenced and controlled by glutamine synthetase [32,33,34]. Glutamine is the first organic nitrogenous molecule that is transaminated to produce other amino acids or N-containing compounds [35]. In non-stressed plants, proline accumulates in vacuoles while under abiotic stresses high proline content is detected in the cytosol [36]. Moreover, under different stresses proline accounts for up to 80% of the total pool of the free amino acids in comparison to less than 5% under normal conditions. This greater accumulation of proline under stress conditions is attributed to enhanced biosynthesis and limited degradation of this molecule [37]. Enzymes involved in proline biosynthesis, including P5CS1, P5CS2 and P5CR, are thought to be localised in the cytosol, while enzymes involved in proline degradation, such as PDH1/ERD5, PDH2, P5CDH and OAT, are localised in mitochondria [38]. Proline transport through the plasma membrane occurs by two super-families transporters. The first group is the amino acid-polyamine-organocation (APC) and the second group is the amino acid transporter family (ATF). Each superfamily is divided into sub-families. ATF family is divided into amino acid permeases (AAPs), lysine, histidine transporters (LHTs), L-proline transporters (ProTs), amino acid/auxin permease (AAAP), γ-aminobutyric acid transporters (GATs), aromatic and neutral amino acid transporters, and amino acid transporter-like proteins. The APC family consists of amino acid/choline transporters, polyamine H+-symporters (PHSs) and cationic amino acid transporters (CATs) [39,40,41].

Eight different amino acid transporters have been identified in *Arabidopsis* [40], among them two encoding specific Pro transporters (ProT1 and ProT2). AAP sub-family and Pro T are expressed during stressful conditions and are responsible for long-distance intercellular transport, which is located at the plasma membrane [15,42]. However, ProT1 was expressed in all organs, but the highest levels were recorded in roots, stems, and flowers in *Arabidopsis* plants under salinity stress. Young flowers showed the highest expression, particularly in floral stalk phloem. Although ProT2 transcripts are found throughout the plant, this expression was strongly induced for nitrogen transport under salinity and drought stress. This is because amino acid transporters are the main mediators of nitrogen distribution in the plant [40,41].

Some evidence indicates that Pro transport increases under abiotic stressed conditions. High proline concentration was reported in the phloem sap of alfalfa under drought stress and active expression of ProT1 and ProT2 was observed in roots [43]. ProT2 was strongly expressed in maize root tips under stress [44]. The results suggest that proline synthesis is more in roots than in shoots. Then, under salt stress, it is transported into the shoot and root tip, while the transport of broad amino acids is suppressed [45]. Under abiotic stress, these transporters can be regulated by both increased gene expression and activity. Based on digital expression analysis, the *SlProT1* and *SlProT2* genes of tomatoes are more active in response to abiotic stress than the others [46]. However, little research has been done on the mechanisms of Pro accumulation and transportation in plants under stress conditions. After biosynthesis intracellular transport occurs between chloroplast, cytosol, and mitochondria. Proline uptake in mitochondria is mediated by many transporters and it is an active transport [45]. The proton uncoupler carbonyl cyanide m-chlorophenylhydrazone (CCCP) inhibits the transport of proline and betaine in sugar beets. Their transport is a pH-dependent process [47]. Application of exogenous proline to leaves may mitigate the adverse effect of stress even when proline transport between root and shoot is limited.

It appears that both ABA-dependent and ABA -independent signaling pathways mediate proline accumulation. ABA-independent signaling involves the activation of SnRK2s or the accumulation of toxic hydrogen peroxide [48,49]. The SnRK2 family, the major regulators of plant response to osmotic stress, are classified into three groups based on their response to ABA. ABA-non-activated kinases belong to the first group, the second group contains kinases that are weakly or not activated by ABA, and the third group includes kinases that are strongly activated by ABA [50].

The primary signaling pathway of proline biosynthesis is mitogen-activated protein kinase (MAPK). Various stimulants modulate the receptor in this cascade. A signal from the cell surface receptor is transduced to DNA by MAPK [51]. The MAP kinase signaling pathway is triggered by signals transmitted from the receptor kinase at the plasma membrane. This signaling pathway also includes MAP kinase kinases (MAP3Ks/MAPKKKs/MEKKs), MAP kinase kinases (MAP2Ks/MAPKKs/MEKs/MKKs) and MAP kinases (MAPKs/MPKs) [52]. During osmotic stress, MAP3Ks phosphorylate and activate SnRK2s. Based on transcriptome analyses, it was found that the genes downstream of these MAP3Ks largely overlap with SnRK2-regulated genes. Thus, MAP3Ks are upstream factors of SnRK2 [53]. ABA Sensitivity and SnRK2 activation were greatly reduced in MAP3Ks knock-out plants. According to these findings, the MAP3Ks clade is necessary for the ABA—and osmotic stress activation of SnRK2 kinases [54]. Since activation of the P5CS1 gene is regulated by ABA signals, ABA is an essential component of signal transduction during stress. Proline accumulates as part of the following process, ABA signals activate the *P5CS1* gene, then stimulated P5CS1 suppresses the *PDH1* genes. during phosphate starvation, family *PHR1* and PHL1 bind to the P1BS motif and induce *P5CS*. Also, they stimulate PDH2. All in all, ABA signals control plant growth and activate several stress-associated genes such as PHL1 and P5CS1 during the stress [28,55].

## 4. The Physiological Function of Proline

Seed germination is a critical stage in the lifecycle of higher plants, affecting yield and crop quality [56]. Several studies found that the expression of genes encoding enzymes of proline metabolism was observed in immature/mature seeds of *Vicia faba*, *Arabidopsis*, *Solanum lycopersicum*, and *Oryza sativa* [57,58,59,60], what suggests that it may be important for generative organ development. In reproductive tissues such as florets, pollens, silicas, and seeds of *Arabidopsis*, proline accounts for about 26% of the total amino acid pool, whereas in vegetative tissues it accounts for only 1 to 3% [61]. In a study conducted on field bean plants, it was found that the ratio of free proline and total free amino acid concentration in different organs is not constant during plant growth. The concentration of free and bound proline was very high in the youngest seeds and pod walls and decreased rapidly during maturation. Venekamp and Koot [57] suggested therefore that free proline has a specific function in the growth of reproductive organs. Funk et al. [58] also demonstrated using the insertion mutants in the P5CS1 and P5CS2 genes that both P5CS and P5CR enzyme activities are necessary for successful sexual reproduction in *Arabidopsis*. Székely et al. [62] confirmed previous studies and found the increased transcription of *P5CS1* and *P5CS2* during *Arabidopsis* embryogenesis. According to Meinke et al. [63], disturbance of P5CR expression resulted in defects in plant embryos and also slowed root emergence suggesting that proline synthesis restores the NADP${}^{+}$ pool and triggers the oxidative pentose phosphate pathway. Mattioli et al. [64] reported that P5CS2 is specifically involved in embryogenesis and cell division, as embryonic growth was arrested in *p5cs2* mutants and accelerated meristem growth by the use of external proline. *P5CS2* is generally more highly expressed in dividing meristematic cells, growing tissue and callus.

There is ample evidence of the significant concentration of proline in flowers and reproductive parts of the plant such as developing seeds, ovules, pollen grains, guard cells, and actively dividing cells [65]. More transcripts of *P5CR* were observed in growing seeds, oocytes, pollen grains, guard cells, and actively dividing cells, which enhanced the recent suggestion that proline plays an important role in plant growth and differentiation throughout the life cycle [66].High-temperature stress disrupted glucose metabolism and proline metabolism during pollen growth, resulting in sterile males in *Lycopersicon esculentum* [67]. In another study, Funck et al. [58] reported that increased expression of *P5CS* is required for pollen growth because higher concentrations of proline in reproductive parts provide nitrogen supply for pollen grain development and fertilization. High proline content resulted in a higher number of seeds per sorghum panicle and a higher number of seeds per ear under stress and non-stress conditions [68,69]. Proline is involved in successful fertility by preventing water wastage in stigma and pollen [65]. It is also suggested as an important factor in attracting pollinators and has a positive effect on the taste of the nectar according to Nepi et al. [70]. Under normal circumstances, most proline is transferred to the reproductive parts of the plant. Proline accumulation in tomato flowers is 60 times higher than in other tissues like leaf, root, and fruit, and 70% of all flower amino acids are restricted to pollen [59,71]. Overexpression of *P5CS* in *Arabidopsis* and tobacco increases proline concentration as a strong signaling molecule that alters floral architecture, accelerates flowering, and increases the number of flowers per plant [61]. Furthermore, there is a positive correlation between proline and stem elongation, with proline providing sufficient energy for cell growth. On the other hand, hydroxyproline-rich glycoproteins are important components of plant cell wall structure. The reduced proline and hydroxyproline content in the cell wall of transgenic *Arabidopsis* confirms this hypothesis [61,72]. Wilson et al. [73] cloned a gene for proline-rich protein (*MtPRP4*) in *Medicago truncatula*. This protein was most abundant in the early development of the nodule meristem, especially in the mature meristem cells of the nodule. These results suggest that *MtPRP4* plays an important role in the initial response of host roots of legumes to *Rhizobium*. Therefore, proline in non-stress conditions can play different roles in different tissues. Since we do not know exactly the molecular events that cause proline to affect flowering and other organs’ development, molecular studies are needed to discover the role of proline during different developmental stages.

## 5. Application of Proline through Seed Priming

Seed priming is an eco-friendly technique that allows plants to respond faster and stronger when exposed to biotic and abiotic stresses [74,75]. Seed priming reflects the natural watering and drying cycle and could be treated as a pre-exposition of seeds under harsh environmental conditions [76]. Priming is a controlled seed rehydration method that stimulates metabolism before germination. These metabolic processes are normally activated in the early stages of germination but inhibit full seed germination. The positive influences of seed priming are due to water uptake and pre-germinative metabolism [77]. There are several priming agents and/or methods including biological priming such as infection by pathogens and colonization by beneficial microorganisms, treatment with natural or synthetic chemicals such as hormonal priming, chemical priming, osmopriming, hydropriming, etc. [78]. Plants can activate the appropriate set of defenses under stress conditions to defend against different stressors by induced resistance [79]. Before a defensive reaction, plants are exposed to significant damage. Seed priming has probably evolved to compensate for this vulnerability, because allows plants to sense environmental cues and prepare them for a rapid and strong response to biotic and abiotic stresses [80]. Many types of molecules such as proline have the potential to act as chemical priming and a useful management tool in agriculture to modulate the adverse influences of abiotic stresses [81,82].

Proline has been recorded as an amino acid with a variety of functions under stress. The main role of this important biomolecule is to protect cellular functions by ROS detoxification, maintaining the integrity of membranes, proteins and intracellular structures [78]. Proline accumulates in high concentrations in response to the various non-living stresses. Cells have been protected from damage by acting proline as both an osmotic agent and a radical scavenger [65]. The plants have been protected from various stresses and also have recovered from stress more rapidly by proline which is mentioned in Table 1 [12,83,84,85,86]. In a study by Demiralay et al. [87], different application modes of exogenous proline such as seed priming, foliar spray and application to rooting medium can enhance drought stress tolerance by increasing the maximum quantum efficiency of PS II, quantum yield of PS II photochemistry, photochemical quenching and electron transport rate. They also mentioned that the root-treated mode is more effective in overcoming the negative effects of short-term drought in maize.

Hua-long et al. [88] reported that soaking seeds with 15 mmol·L${}^{-1}$ and 30 mmol·L${}^{-1}$ proline enhanced rice germination under salt conditions by improving the amylase activities relative germination energy and relative germination rate. They concluded that pre-treatment of seeds with proline effectively alleviated the negative effects of salinity on seed germination. In another study by Singh et al. [85] on rice exposed to salt stress pre-treatment with proline significantly increased the germination, seed vigor index and α-amylase activity which is the key enzyme in hydrolyzing starch for seed germination.

Also, proline priming is effective and stimulates cellular activities. Proline priming improved antioxidant enzyme activities and photosynthetic pigments in salt-stressed wheat plants. Although salinity caused physiological disturbances through enhanced production of reactive oxygen species, the increase in the antioxidant activities of superoxide dismutase (SOD), peroxidase (POD), and catalase (CAT) were observed following priming with 4 and 8 mM proline for 8h in different wheat cultivars [86]. Agami [83] studied the effect of barley seed soaking in proline. Results showed that the length of shoot, the number of leaves, leaf area per plant, plant fresh and dry masses, the content of chlorophyll Chl *a*, Chl *b* and carotenoids were significantly reduced by 100 or 200 mM NaCl but electrolyte leakage, proline content, activities of antioxidant enzymes (SOD, CAT and non-specific peroxidase (POX)) increased under salinity stress. Seed soaking with proline in the absence or presence of salinity stress increased the leaf thickness and caused a marked variation in antioxidants enzymes. In conclusion, inhibition of oxidative damage in barely cells through increasing the activity of the antioxidant system by application of exogenous proline protected the photosynthetic machinery and increased the tolerance to the salt stress. Rady and Hemida [89] showed that soaking maize seeds in proline improved seedling growth in stressed and non-stressed conditions. The combined use of proline, glutathione and ascorbic acid increased the relative water content (RWC), membrane stability index (MSI), and minimized electrolyte leakage (EL) of maize seeds in control conditions and increased protein concentrations and activities of antioxidant enzymes (SOD and glutathione peroxidase (GPOX)) and decreased activity of CAT in NaCl-stressed plants. Although IAA, GA3and zeatin decreased in maize seedlings under salinity stress, authors reported that seedlings pre-treated with antioxidants compounds increased them significantly and reduced ABA concentration. Thus, antioxidants pre-treatment stimulate seedling cells to grow rapidly under stress by increasing cell elongation and cell division through changes in phytohormones.

Proline seed priming alleviated cadmium toxicity and improved growth attributes in cowpea through improving chlorophyll value, stomatal conductance, RWC, antioxidant enzyme activities, preventing membrane lipid peroxidation and reducing Cd uptake and translocation [12]. Karalija and Selović [84] tested the effect of maize seed priming with proline under cadmium stress. In this study, growth characteristics, proline and sugar content, as well as antioxidant activity were studied. Although proline priming increased osmoprotectant molecules such as proline and sugars, antioxidant activity was not sufficient to overcome Cd toxicity, because it cannot control hydroxyl radical production induced by cadmium. The main mitigating mechanism in proline-primed maize seeds is the scavenging of ROS by proline and soluble sugars as non-enzymatic components. In another study by Yaqoob et al. [90], seed priming and spraying of proline on quinoa under low-temperature stress significantly improved plant growth, photosynthetic pigments, osmolites) proline, ascorbic acid (total free amino acids, phenolics, total soluble carbohydrates, activities of antioxidant enzymes (SOD, POD, and CAT) but decreased malondialdehyde (MDA) content. The results show that these different abiotic stressors trigger similar types of signaling pathways and cellular responses. Seed priming promotes these signaling pathways earlier and improves plant defense reactions. Thus, for better plant production, seed priming is a realistic and effective choice.

## 6. Foliar Application of Proline

Application of exogenous proline to plants exposed to stress increases growth and other physiological characteristics of plants [15]. One of the widely used methods of proline application is a foliar spray on the shoots ts. In the following chapter, the effects of proline application as a foliar spray are discussed regarding selected abiotic stresses.

### 6.1. Effect of Proline Foliar Application in Drought Stress

Drought is one of the main non-biological stressors that threaten food security by reducing crop yields. The drought that is often linked with other major abiotic stresses has become a challenging issue due to climate change, increasing demand for water and decreasing water supply. Drought stress affects plant performance through physical damage, molecular changes, physiological and biochemical disorders [13,91]. The useful effect of proline foliar application on plants exposed to drought stress is shown in Table 2. AlKahtani et al. [92] studied the effect of proline application on sugar beet under drought stress conditions. Stress symptoms such as ROS production, electrolyte leakage and lipid peroxidation were increased under stress conditions. Application of exogenous proline increased proline content, total phenolic compounds, and antioxidant enzyme activity which reduced measured stress symptoms. In the study, Elewa et al. [13] reported a significant reduction in endogenous auxin (IAA), grain yield and grain nutritional value by omitting two irrigations. The quantitative and qualitative yield of quinoa seeds and antioxidant activity increased with increasing the concentration of exogenous proline. The chlorophyll content of leaves is one of the most important criteria for determining the environmental pressure on plants, which decreases under stress and consequently reduces light absorption by the plant [91].

Farooq et al. [93] reported that the use of proline as an osmotic protector against water deficiency caused the accumulation of high content of chlorophyll, proline, glycine betaine, and total soluble phenols in the wheat plant. Proline accumulation during drought stress was associated with increased proline biosynthesis precursors, such as glutamic acid, ornithine, and arginine [78]. Merwad et al. [94] noticed in cowpea exposed to drought stress that proline, silicon or methionine foliar application increased growth and yield characteristics, leaf chlorophylls *a* and textitb, total carotenoids, shoot and seed nutrients, relative water content, and enzymatic antioxidant activities and reduced levels of MDA and H${}_{2}$O${}_{2}$. Dehydration is known to induce expression of the gene encoding proline carboxylate synthetase, leading to increased PCS activity and proline content [45].

The key enzymes of proline biosynthesis are regulated by the exogenous proline, which can act as an antioxidant or osmolyte to decrease the negative effects of abiotic stress [92]. However, the true mechanism of regulation of genes related to proline metabolism and signaling mechanisms is still unknown. Osmoprotectants are involved in plant tolerance to drought stress by the regulation of metabolic processes, including ion transport. For this reason, Ali et al. [95] investigated the effects of proline foliar application on the uptake of some essential nutrients in maize during vegetative growth. Water stress decreased four mineral nutrients content in aerial parts and roots of maize. However, the external application of proline successfully alleviated the negative impacts of water stress by increasing the uptake of K${}^{+}$, Ca${}^{2+}$, N and P.

### 6.2. Effect of Proline Foliar Application in Salinity Stress

Salinity stress as one of the most important abiotic stresses in many parts of the world limits the growth and development of plants. About 7% of terrestrial land is influenced by salinity [96]. Factors causing this stress include soil salinity, irrigation with saltwater, and poor drainage [97]. Increasing concentration of salt in the soil makes it more difficult to absorb water from roots and the accumulation of salt inside the plant can also be toxic [98,99]. Toxicity due to ions and osmotic stress cause nutrient imbalance in plants. Plants under stress make osmotic regulation by accumulating K${}^{+}$ and Ca${}^{2+}$ and removing Na${}^{+}$ and Cl${}^{-}$ ions, which cause ion toxicity [14,18,52,100]. Some scientific reports on foliar application of proline under salinity stress are listed in Table 3.

Siddiqui et al. [101] studied different wheat cultivars under salt stress and reported that plant tolerance to salt stress depends on ion toxicity and oxidative stress. Therefore, reducing the Na${}^{+}$:K${}^{+}$ ratio and oxidative stress and increasing the content of the internal proline improve growth and yield. Several compatible solutes such as proline, betaine, trehalose, ectoine and polyols are accumulated in the cytosol in saline conditions. Proline as an essential amino acid is also a vital osmotic compound that protects plants from oxidative stress [102]. Patel et al. [103] reported in their review study that the use of exogenous metabolites without genome manipulation could be considered a promising approach to improve plant tolerance and reduce the effects of salt stress. Increased growth, photosynthesis and yield parameters due to proline application were reported under non-stress conditions [99]. Numerous studies confirm the positive effect of proline application in improving the plant’s enzymatic antioxidant defense system against salinity stress [14,18,104].

In *Brassica juncea* L. foliar application of proline neutralized the negative effects of low salinity levels by improving the antioxidant capacity [99]. Foliar application of proline in chili pepper seedlings subjected to salt stress resulted in improvements in morphological and physiological traits, including increases in stem and root length, dry and fresh plant mass, photosynthetic rate, transpiration rate, and antioxidant enzyme activity [18]. Qirat et al. [14], observed improvement in the photosynthetic system, gas exchange, chlorophyll fluorescence, antioxidant system, and finally growth of carrot plants in the presence of exogenous proline and salt stress. Other studies have reported the positive effect of exogenous proline application on red pepper, rapeseed, common sainfoin and olive under salinity conditions [100,105,106,107]. Abdelhamid et al. [104] recommended 5 mM proline foliar application to decrease the salinity impacts on common beans. According to their findings, the use of proline increased the concentrations of carotenoids, ascorbic acid, endogenous proline, phosphorus and potassium in plants exposed to stress and decreased the concentration of Na${}^{+}$ ions. Shaddad [108] also reported that foliar application of radish with proline through osmotic regulation reduced the salinity effect. Salinity stress reduced eggplant growth by reducing net CO${}_{2}$ assimilation rate, water use efficiency, photosystem II efficiency, and K${}^{+}$ and Ca${}^{2+}$ ions in shoots and roots.

Proline application affected only shoot fresh weight and did not moderate the effects of salinity stress [109]. The findings of Mahboob et al. [110] confirmed the effectiveness of proline in increasing salt tolerance in wheat plants. Exogenous proline counteracted salt stress by improving physiological and biochemical processes, including increasing proline, glycine-betaine, total soluble sugar, total phenolic content, chlorophyll *a* and *b* content, and adjusting the K${}^{+}$:Na${}^{+}$ ratio. The results of another study on the effect of proline on the modulation of seawater stress showed that although the external application of proline at a concentration of 25 mM somewhat reduced seawater-induced toxicity in broad bean plants, treatment with proline at a concentration of 50 mM was toxic [111].

### 6.3. Effect of Proline Foliar Application in Temperature Stress

The effects of high and low-temperature stress vary according to the duration, intensity, and plant life stage [112]. Temperatures above the desired temperature have negative effects on the plant. They stimulate the fall of buds, flowers, and fruits and reduce the production of seeds and fruits [113]. The transfer of photosynthetic material to the reproductive organs is disrupted, making fruiting more difficult. Other effects of heat stress include twisting, premature flowering of the plant, sunburn, denaturation of proteins, increased membrane fluidity, and impaired plant growth and development [114]. As mentioned in Table 4, High-temperature stress in heat-sensitive/tolerant genotypes of okra showed higher levels of antioxidant enzymes (SOD, CAT, and POX) in heat-tolerant genotypes. Foliar application of 2.5 mM proline showed maximum levels of proline, glycine betaine, free amino acids, and chlorophyll in leaves of stressed plants [115].

Orsini et al. [19], also reported effective foliar application of proline up to 5 μM to improve lettuce yield in response to temperature and salinity stress. Priya et al. [20], exposed the mung bean plants to high-temperature stress at the beginning of flowering to investigate the effect of exogenous proline application on reproductive organs and flower reproductive performance. Proline treatment, especially at high-temperature stress, improved endogenous proline in the vegetative and reproductive parts, pollen fertility, stigma, and finally egg function. Also, exogenous proline had a significant effect on the yield components of mung bean, including pod number, pod weight, and seed weight per pod, by reducing damage to the photosynthetic system and improving leaf water status. Confirming the previous report, proline also acts as a nitrogen and carbon source, improving the growth and regeneration of *Vigna radiata* seedlings under cold stress [116].

Low-temperature stress can occur in the form of cold stress or frost stress [112]. Cold stress causes physical disturbances in cells and affects physiological activities, and various growth processes. In general, physical injuries caused by cold stress include discoloration, drying, and aging due to ethylene production. Also, several changes to ion homeostasis by increased cellular Ca${}^{+}$ content, accumulation of ROS, protein denaturation, and solute precipitation. Eventually, cold stress can lead to cell death [15]. Nawaz et al. [117] reported that the removal of proline-rich proteins in rice (OsPRP1) leads to cold sensitivity at the seedling stage. Different concentrations of proline foliar application under cold stress in three citrus species increased flavonoids, oxalic acid, citric acid, ascorbic acid, and antioxidant enzymes such as ascorbate peroxidase and catalase. In contrast, tartaric acid and lipoxygenase activity decreased with increasing temperature and exogenous proline content [118]. Koç [119] reported that the increase of proline and apoplastic protein by proline and salicylic acid reduced the adverse effects of cold stress on pepper callus.

### 6.4. Effect of Proline Foliar Application in Heavy Metals Stress

Increasing the concentration of heavy metals such as lead, copper, nickel, and other heavy metals in water, soil, and air can have harmful effects on the health of all living organisms due to their negative impact on the entire ecosystem [112]. The most important sources of these pollutants in most parts of the world are mines, industrial effluents, chemical fertilizers, and pesticides. Toxic heavy metals such as lead, cobalt, and cadmium are non-biodegradable, can accumulate in living organisms and can have deleterious effects on biological systems [120]. The penetration of heavy metals into the plant decreases growth and disrupts cellular metabolism by damaging cell membranes and DNA structure, altering enzyme properties and disrupting cellular functions. Finally, it negatively affects important processes such as water transfer, mitochondrial oxidative phosphorylation, photosynthesis, and chlorophyll content [80]. Plants use physiological and molecular mechanisms such as selective adsorption of metals, entrapment by the root cell walls, chelation with phytochelatins, metallothioneins, amino acids and glutathione, accumulation of metals in vacuoles, and antioxidant enzymes to neutralize antioxidant activity [112].

The high content proline in the plant is a reaction to the induction of stress, and the high production of this amino acid can cause the plant to tolerate stress [121]. In the presence of heavy metals, proline acts as a metal chelator, an antioxidant defense molecule, and a messenger molecule [15]. Elevated levels of proline have been found in response to stress from various metals and in various plants such as chickpea in cadmium, pea in nickel, trifoliate orange in aluminum, and olive tree in cadmium toxicity [122,123,124,125]. Table 5 shows the effects of the proline application in modulating the stress of the heavy metal. Alyemeni et al. [123] studied the response of chickpea in the presence of cadmium at concentrations of 0, 25, 50, and 100 mg cadmium per kg soil and foliar application of 20 mM proline. Nodulation, hemoglobin, carbohydrate, leaf and root nitrogen content, the enzymatic activity of nitrogenase, nitrate reductase, glutamine synthetase, glutamate synthase, and glutamate dehydrogenase increased with proline application. However, the application of proline was not effective at the highest cadmium concentrations and the studied characteristics improved at cadmium 25 mg/kg compared to control but there was no significant difference at cadmium 50 mg/kg compared to control.

**Figure 2:**
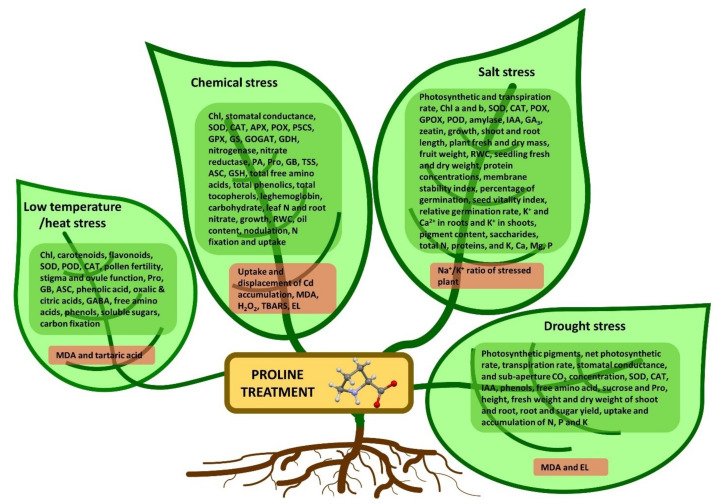
The summarized physiological and biochemical changes observed under the influence of exogenous proline that have beneficial effects on the tolerance of stress factors in cultivated plants. Up-regulated processes and/or biochemicals are marked in green, down-regulated are marked in red. For detailed information refer to text.

In another study pure proline and proline extracted from Lolium perenne L. leaf were examined on chickpea plants under salinity and nickel stresses. In plants under stress free radicals of oxygen, osmolytes (proline, glycine-betaine, total free amino acids), total soluble carbohydrates, total phenols, lipid peroxidation and electrolyte leakage increased significantly. In contrast, total chlorophyll content, photosynthetic activity, stomatal conductance, relative water content (RWC), and membrane stability index decreased. The application of pure and natural proline alleviated the toxicity of the stress by preventing lipid peroxidation and electrolyte leakage and increasing osmolyte content and polyamine biosynthetic enzyme activity [122]. In the study of Singh et al. [121], eggplant seedlings were treated with proline in hydroponic culture containing arsenate. The activity of P5CS, which is a key enzyme for proline biosynthesis, was increased in proline-treated seedlings and PDH activity was inhibited under arsenate stress. The authors concluded that proline metabolism plays a key role in regulating arsenate accumulation and improving the antioxidant system of eggplant seedlings. Yan et al. [125], tested the proline-containing nutrient solution (0.2 mM) on trifoliate orange in a boron-deficient and aluminum-toxic condition. Since the use of proline reduced the damage caused by boron deficiencybut increased the toxicity of aluminum limiting the growth of the plant, the use of external proline was considered detrimental to tomato seedlings. The toxicity of different levels of selenium (1, 2, 4, and 6 ppm) and the effect of proline application was studied on bean seedlings in hydroponic culture. Low levels of selenium improved root and shoot growth by increasing chlorophyll content and leaf water content, while high levels of selenium significantly reduced growth by damaging cell membranes and oxidative injury. Application of proline in a culture medium also reduced selenium stress damage by increasing the content of endogenous proline and enzymatic and non-enzymatic antioxidants [126]. Zouari et al. [124], used proline (10 and 20 mM) to irrigate olive trees under cadmium stress. Exogenous proline increased antioxidant enzyme activity, photosynthetic activity, and finally plant growth and fruit oil content in trees under cadmium stress. The beneficial function of proline in overcoming the effects of abiotic stresses on plants based on the studies of many researchers has been summarized in Figure 2.

## 7. Conclusions and Future Perspective

One of the most important challenges for plant physiologists is to find the right solutions and reduce the effects of stress. The results of numerous studies show that the use of exogenous proline can mitigate the damage caused by environmental stress in plants. Proline accumulates as a proteogenic amino acid in plants under both stress and normal physiological conditions. Proline is also known in plants as an osmotic and energy supplier, ROS scavenger, and stress reliever. The balance between biosynthesis and proline degradation determines its role as an osmotic protector or growth signal. The relation between proline content and abiotic stress tolerance in plants is still not clearly understood. However, plant researchers agree that proline accumulation is beneficial to plants, especially after recovery from stress. Proline affects plant growth and production via its metabolic signal-mediating function. Further research into the factors that regulate the expression of enzymes involved in proline synthesis and degradation will be useful for improving stress tolerance in plants. Not only exogenous application of proline could be used for induction of stress response, but also molecular engineering modulating genes expression through gene modifications or gene editing. However based on regulations, which restrict applications of such organisms in particular country development of non-invasive techniques seems to be rationale choice. It is also necessary for a better understanding of the stress-related aspects in the regulatory network that controls plant responses to the stress. Although the application of exogenous proline is an effective means of reducing the adverse effects of stress, the effect of proline on plants depends on the species, the growth stage, the timing and method of application, and the proline concentration. That is why the advanced study on proline metabolism in stress response should be attentive. The obtained results and achievements are important for farming in stressed areas and may extend attempts to develop more efficient crop stress management system in the near future.

## Figures and Tables

**Table 1:** Published scientific reports of seed priming with proline to enhance stress tolerance.

Plant	Stress	Observed Effects	Reference
*Chenopodium quinoa* Willd.	Low-temperature (Seeds kept at 4 °C for 20 h before sowing)	Plant growth improved by increasing the content of chlorophyll, carotenoids, proline, ascorbic acid, total free amino acids, phenols, total soluble sugars, the activity of SOD, POD and CAT and reducing MDA	Yaqoob et al., 2019 [90]
*Hordeum vulgare*	Salinity (0, 100 and 200 mM NaCl)	Proline treatment decreased the salinity impacts by increasing the activity of antioxidant enzymes (SOD, CAT and POX) and affecting leaf anatomy	Agami, 2014 [83]
*Oryza sativa* L.	Salinity (0 and 100 mM NaCl)	Rice germination, relative germination rate and, the amylase activities under stress heve been increased	Hua-long et al., 2014 [88]
*Oryza sativa* L.	Salinity (0, 50, 100, 150 and 200 mM NaCl)	The percentage of germination, seed vitality index, and alpha-amylase activity significantly increased	Singh et al., 2018 [85]
*Triticum aestivum* L.	Salinity (0 and 150 mM NaCl)	The growth and activity of the antioxidant enzyme improved, which depends in part on the plant’s ability to absorb sodium and distribute it to the roots	Shafiq et al., 2018 [86]
*Vigna unguiculata* L.	Cadmium (0 and 100 μM cadmium chloride)	The uptake and displacement of cadmium and MDA reduced, the amount of chlorophyll, stomatal conductance, and RWC, the activity of SOD, CAT and APX increased	Sadeghipour, 2020 [12]
*Zea mays* L.	Drought (0 and −0.5 MPa by polyethylene glycol (PEG 6000))	Root treatment by 1 mM PRO were more effective than foliar spraying on water potential, chlorophyll content, proline level, net photosynthetic rate, transpiration rate, stomatal conductance, and sub-aperture $CO_{2}$ concentration	Demiralay et al., 2017 [87]
*Zea mays* L.	Salinity (0 and 100 mM NaCl)	IAA, GA3, zeatin, SOD, GPOX Activities, protein concentrations, RWC, membrane stability index increased and ABA and CAT decreased under salt stress	Rady and Hemida, 2016 [89]
*Zea mays* L.	Cadmium (0, 5 and 50 μM Cd${}^{2+}$ ions obtained from CdCl${}_{2}\cdot$2.5H${}_{2}$O)	Improving defensive mechanisms, like proline and sugar synthesis and increasing Cd accumulation induced by proline priming	Karalija and Selović, 2018 [84]

**Table 2:** Published scientific reports of foliar application of proline to enhance drought stress tolerance.

Plant	Stress	Observed Effects	Reference
*Beta vulgaris*	Normal irrigation and 50% field capacity	The application of silicon and proline increased CAT and SOD activity, root and sugar yield, sucrose%, Chl content, phenolic compounds and reduced RWC, MDA and EL	Alkahtani et al., 2021 [92]
*Chenopodium quinoa*	Normal irrigation and skipping two irrigation times at 45 and 52 days after sowing	Exogenous proline has the greatest effect on photosynthetic pigments, IAA, phenols, free amino acid and proline content, plant height, fresh and dry weight of shoot and root	Elewa et al., 2017 [13]
*Triticum aestivum* L.	Normal irrigation and drought at 35% water holding capacity	Application of proline at 150 ppm and GABA at 100 ppm were recommended as the most effective concentrations on chl, proline, glycine betaine and total soluble phenolics contents and reduced MDA	Farooq et al., 2017 [93]
*Zea mays* L.	Full field and 60% field capacity	Application of proline in modulating drought stress by promoting the uptake and accumulation of N, P and K${}^{+}$	Ali et al., 2008 [95]

**Table 3:** Published scientific reports of foliar application of proline to enhance salt stress tolerance.

Plant	Stress	Observed Effects	Reference
*Brassica juncea*	2.8, 4.2 and 5.6 ds·m${}^{-1}$ NaCl	Although proline (20 mM) improved yield in plants without stress and under mild stress, it was not effective at high salinity stress levels	Wani et al., 2016 [99]
*Capsicum annuum* L.	0 and 50 mM NaCl	The greatest improvement in SOD and CAT activities, photosynthetic and transpiration rate, shoot and root length, plant fresh and dry mass was obtained at a concentration of 0.8 mM proline	Butt et al., 2016 [18]
*Capsicum annuum* L.	0.6, 4.04 and 6.11 ds·m${}^{-1}$	Proline and L-tryptophan treatment improved shoot dry weight, fruit weight, RWC, Photosynthetic pigments and decreased the Na${}^{+}$/K${}^{+}$ ratio	Jamil et al., 2018 [100]
*Daucus carota* L.	0 and 150 NaCl	Proline application increased the activity of SOD, POD, free proline content, and K${}^{+}$ and Ca${}^{+}$ in roots and K${}^{+}$ in shoots	Qirat et al., 2018 [14]
*Phaseolus vulgaris* L.	1.84, 6.03 and 8.97 ds·m${}^{-1}$	SOD, CAT and POD, carotenoids, ascorbic acid, endogenous proline, the concentrations of P and the K${}^{+}$:Na${}^{+}$ ratio under salt stress by exogenous proline.	Abdelhamid et al., 2013 [104]
*Raphanus sativus*	0, 40, 80, 120 and 160 mM NaC1	Transpiration rate, stomatal frequency, pigment content, saccharides, total nitrogen, proteins, and some nutrients (K, Ca, Mg, P) improved at low and moderate salt stress	Shaddad, 1990 [108]
*Solanum melongena* L.	0 and 150 mM NaC1	Proline spraying did not affect the modulation of salinity stress impacts	Shahbaz et al., 2013 [109]
*Triticum aestivum* L.	0, 60 and 120 Mm NaCl	Root and shoot length, seedling fresh and dry weight, Chl *a* and *b* content, TSS, Pro, GB, TPC, and K${}^{+}$ content, and K${}^{+}$:Na${}^{+}$ ratio improved in stressed plants	Mahboob et al., 2016 [109]
*Vicia faba*	0.23 ds·m${}^{-1}$ tap water, 3.13 and 6.25 ds·m${}^{-1}$ diluted seawater	Photosynthetic pigments, N, P, K${}^{+}$, and Ca${}^{+2}$ content, K${}^{+}$:Na${}^{+}$ ratio, and total soluble carbohydrates increased, while Na${}^{+}$, Cl${}^{-}$, phenolic content, free amino acids, and proline reduced	Dawood et al., 2014 [111]

**Table 4:** Published scientific reports of foliar application of proline to enhance temperature stress tolerance.

Plant	Stress	Observed Effects	Reference
*Abelmoschus esculentus* L.	Heat (Gradually increasing the temperature of the growth chamber from 28/22 °C to 45/35 °C (day/night))	Shoot length, leaves per plant, SOD, POD and CAT activity, leaf Pro, GB, total free amino acids, and Chl content increased	Hussain et al., 2021 [115]
*Citrus reticulata*/ *C. sinensis*/ *C. paradisi*	low-temperature (shoots exposed to temperatures 1, −1 and −3 °C for three hours)	The amount of phenolic acid, flavonoids, oxalic, citric, and ascorbic acids, gamma-aminobutyric acid, endogenous proline, APX, and CAT increased and tartaric acid reduced	Mohammadrezakhani et al., 2019 [118]
*Lactuca sativa*	Salinity/Light/Heat (0 and 15 mM NaCl. Transparent or white film Late spring, summer and fall)	The combine both white covering film and 5 μM proline application under the salinity of 15 mM, improved efficient preservation of plant growth, photosynthetic rate, Chl content and yield	Orsini et al., 2018 [19]
*Capsicum annuum* L.	Cold (Callus tissues were developed at 4, 8, 16 and 24 °C)	The maximum amount of endogenous proline and apoplastic protein at the lowest temperature (4 °C) was observed by the simultaneous application of proline (24 mM) and SA (0.25 mM)	Koç, 2013 [119]
*Vigna radiata* L.	high-temperature (30-day old plants were exposed to 35/23 °C, 40/28 °C and 45/33 °C (day/night))	Pollen fertility, stigma and ovule function, carbon fixation, and assimilative capacity of heat-stressed mung bean plants were improved	Priya et al., 2019 [20]

**Table 5:** Published scientific reports of exogenous application of proline to enhance heavy metals stress tolerance.

Plant	Stress	Application	Observed Effects	Reference
*Cicer arietinum* L.	Cadmium (0, 25, 50 or 100 mg of cadmium per kg of soil)	Foliar	Nodulation, nitrogen fixation and uptake, leghemoglobin, carbohydrate, content of nitrogen in leaf and nitrate in root, the activity of nitrogenase, nitrate reductase, GS, GOGAT and GDH increased	Alyemeni et al., 2016 [123]
*Olea europaea* L. cv Chemlali	Cadmium (0, 10 and 30 mg CdCl${}_{2}$ per kg of soil)	Irrigation	The concentration of proline 20 mM enhancing oil and proline content, SOD, CAT, APX and GPX and reducing H${}_{2}$O${}_{2}$, TBARS and EL	Zouari et al., 2016 [124]
*Phaseolus vulgaris* L.	Selenium (0, 1, 2, 4, and 6 ppm)	Culture medium	Chl and endogenous proline content, RWC, SOD, CAT, APX activity, ASC and GSH content increased and H${}_{2}$O${}_{2}$ reduced	Aggarwal et al., 2011 [126]
*Pisum sativum* L.	Salinity/Nickel (0 and 100 mM NaCl 0 and 100 μM Nickel)	Foliar	Pure proline and natural proline (*Lolium perenne* L. leaf extract) detoxified the stress caused by NiCl${}_{2}$ and/or NaCl. LP extract can be used as a cheap source of proline to enhance growth, polyamine metabolism, photosynthetic activity, RWC, organic osmolytes.	Shahid et al., 2014 [122]
*Poncirus trifoliata*	Boron deficiency/ aluminum toxicity (0.1 μM B, 300 μM Al and 0.1 μM B + 300 μM Al)	Nutrient solution	Exogenous proline enhanced cellulose and protein content. In contrast, higher MDA and H${}_{2}$O${}_{2}$ contents inhibited the plant growth	Yan et al., 2020 [125]
*Solanum melongena* L.	Arsenate (0, 5 and 25 μM)	Seedling treatment	Exogenous proline decreased the accumulation of As and attenuated its toxicity by increasing the activity of SOD, CAT, POX, and P5CS as well as endogenous Proine	Singh et al., 2015 [121]
